# Capture of emotional responses under a simulated earthquake experience using near-infrared spectroscopy and virtual reality

**DOI:** 10.1371/journal.pone.0304107

**Published:** 2024-05-23

**Authors:** Hikari Otsuka, Sayaka Okahashi, Hirotake Ishii, Wataru Asaba, Chang Liu, Goshiro Yamamoto, Akitoshi Seiyama

**Affiliations:** 1 Graduate School of Medicine, Kyoto University, Kyoto, Japan; 2 Center for Gerontology and Social Science, National Center for Geriatrics and Gerontology, Obu, Japan; 3 Creative Design & Data Science Center, Akita International University, Akita, Japan; 4 Graduate School of Energy Science, Kyoto University, Kyoto, Japan; Chiba Daigaku, JAPAN

## Abstract

**Aim:**

In a previous study, we reported that watching two-dimensional videos of earthquakes significantly reduced sympathetic nerve activity in healthy young adults. In the present study, we aimed to investigate the emotional responses to earthquakes using immersive virtual reality (VR), which can provide a more realistic experience.

**Methods:**

In total, 24 healthy young adults (12 males, 21.4 ± 0.2 years old) participated. Participants were required to watch earthquake and neutral videos while wearing a head-mounted display and near-infrared spectroscopy (NIRS), during which physiological signals, including pulse rate and cerebral blood flow (CBF) in the dorsolateral prefrontal cortex, were measured. We also analyzed changes in sympathetic and parasympathetic indices and obtained seven emotion ratings: valence, arousal, dominance, fear, astonishment, anxiety, and panic.

**Results:**

The VR earthquake videos evoked negative subjective emotions, and the pulse rate significantly decreased. Sympathetic nerve activity tended to decrease, whereas CBF in the left prefrontal cortex showed a slight increase, although this was not significant.

**Conclusions:**

This study showed that measurements combined with NIRS and immersive VR have the potential to capture emotional responses to different stimuli.

## Introduction

When natural disasters such as earthquakes and floods occur, people must evacuate calmly, even though they may be emotionally distressed. Negative emotions (e.g., fear, astonishment, and anxiety) are often elicited in such situations [1]. The degree of fear when encountering an earthquake is related to personal factors, such as gender, age, and the scale of perceived damage [2]. Strong emotional arousal can cognitively disturb thinking calmly and cognitive decision-making [3]. However, the relationship between emotional responses and brain activity during natural disasters remains unclear. Thus, it is important to clarify subjective/objective emotional reactions when encountering disasters, which could be a milestone in designing individualized efficient support in this era of increasing global natural disasters.

Previously, we studied emotional reactions in healthy young adults using earthquake-related videos and recorded physiological indices (e.g., prefrontal cortex blood flow changes) using near-infrared spectroscopy (NIRS) [4]. We found that the sympathetic index significantly decreased under the earthquake video-watching condition, which evoked negative emotions, compared with the neutral video-watching condition. Emotional responses to different stimuli (i.e., earthquake vs. neutral moving images) were also obtained. However, there were some limitations in the experimental setting, in that the two-dimensional (2D) images on the screen might not be realistic enough visually and auditorily. Therefore, we plan to provide a more realistic disaster experience and sufficient emotional arousal using immersive virtual reality (VR).

It is reported that the prefrontal cortex blood flow increased under the immersive nature of images and presentation of various audiovisual stimuli in a virtual space with a greater sense of reality [5]. Related studies have reported multiple NIRS measurements in immersive VR experiences focusing on cognitive functions [6, 7]. However, there are no reports on the emotional/physiological responses to disaster experiences using VR.

In this study, we investigated the human emotional reactions to earthquakes using immersive VR. Specifically, we focused on the changes in cerebral blood flow (CBF) and pulse rate, compared the indexes between the earthquake and neutral videos, and then discussed the differences from the results of our previous study using 2D videos [4]. We hypothesized that the VR earthquake experience will evoke more negative emotions, lower pulse rate, and sympathetic activity and increase CBF in the right prefrontal cortex compared with screen-based video viewing. By clarifying the changes in physiological indices during the simulation of a specific scene, such as an earthquake, this study contributes to the understanding of emotional reactions when encountering an actual earthquake.

## Materials and methods

### Participants

In total, 24 healthy young adults (12 males and 12 females, aged 21.4 ± 0.2 years) participated in the study. Using G*Power 3.1.9.2, pre-analysis for the required minimum sample size resulted in 15 persons, assuming effect size = 0.80, α error probability = 0.05, and power (1-β error) = 0.80. The eligibility criteria were as follows: no history of visual, hearing, verbal comprehension, or mental disorders that might interfere with the performance of the tasks in this study and had never experienced VR sickness prior to this experiment. In addition, the Impact of Event Scale-Revised (IES-R: Japanese version of the revised Impact of Event Scale) was conducted to confirm the absence of prior trauma to earthquake, and individuals with a cutoff value of 24 points or less were included.

This study was approved by the Kyoto University Medical Ethics Committee (R2855). All participants were fully informed before the beginning of the experiment, and informed consent was obtained through both oral and written consent.

### Visual stimulation

Two types of videos were used, earthquake and neutral, described as follows. They were presented via an Oculus Rift head-mounted display (HMD).

#### Earthquake video

**Fig 1:**
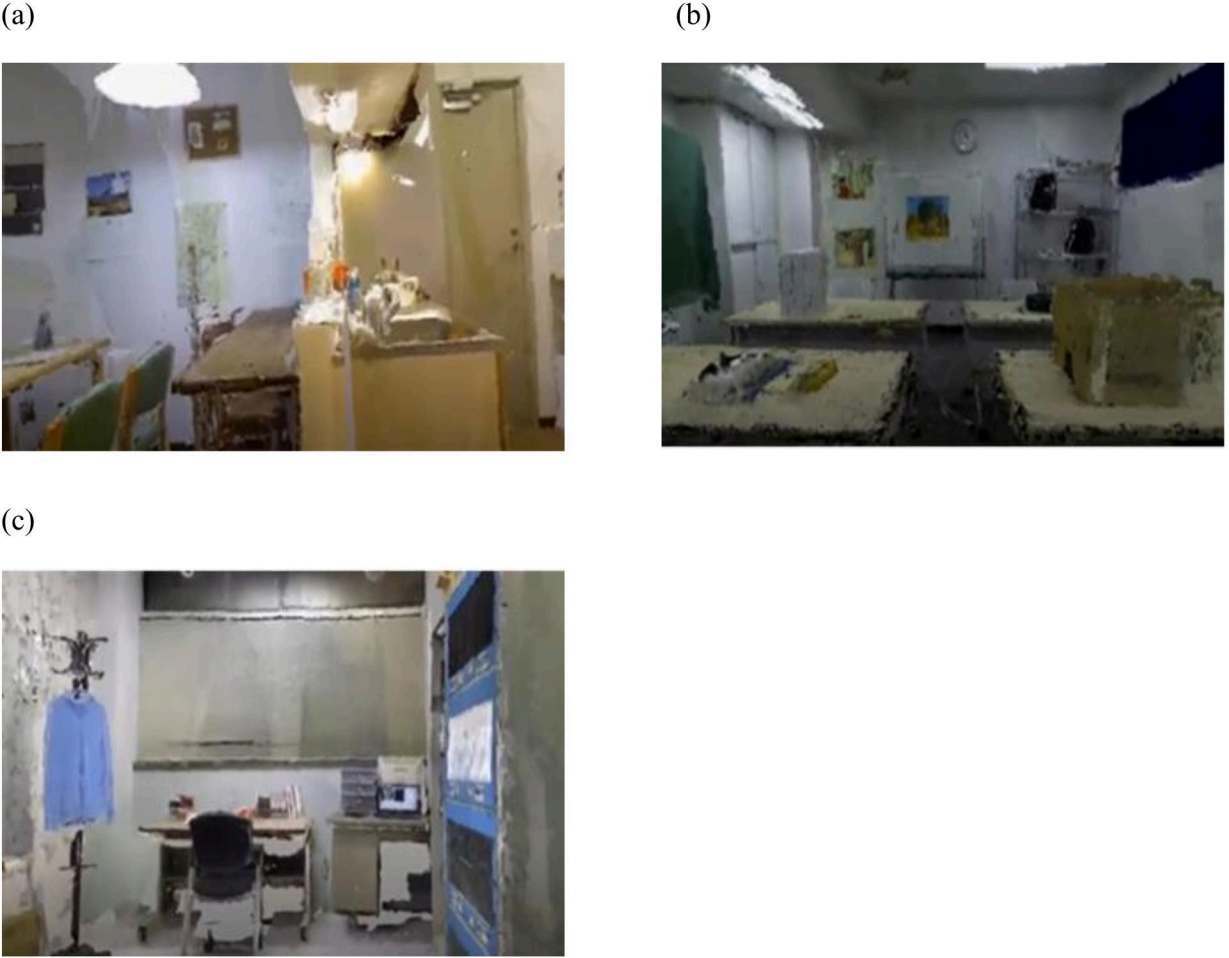
Sample images of the VR earthquake videos. (a) Living room, (b) conference room, and (c) laboratory videos were created using a three-dimensional reconstruction system [8, 9].

An environment for experiencing an earthquake in an immersive VR space was constructed based on a three-dimensional (3D) reconstruction system [8, 9] that captures an indoor environment with an RGB-D camera and reconstructs a 3D environment from the acquired point clouds. In this study, three common environments for the participants: a living room (Fig 1(A)), conference room (Fig 1(B)), and laboratory office (Fig 1(C)), were selected for the content of earthquake videos.

The VR earthquake videos were displayed for 100 s to evoke emotional changes in the event of an earthquake that was closer to reality. They were constructed for experimental use by intervening with visual elements, such as the resolution and behavior of objects and scenes, and auditory elements, such as the rumbling and collision sounds of objects [8, 9].

#### Neutral video

As a control condition for earthquake images, two 100-s neutral video clips were created by adding pink noise to images of natural scenery [10]. The environments included rivers and grasslands in the neutral image; each neutral image is a moving image containing a waterfall flowing grass and trees rustling in the wind. Pink noise, characterized by a soft and pleasant sound whose energy is inversely proportional to its frequency, with strong lows and weak highs, was used to control the auditory stimulus conditions in the seismic video.

### Procedure

**Fig 2:**
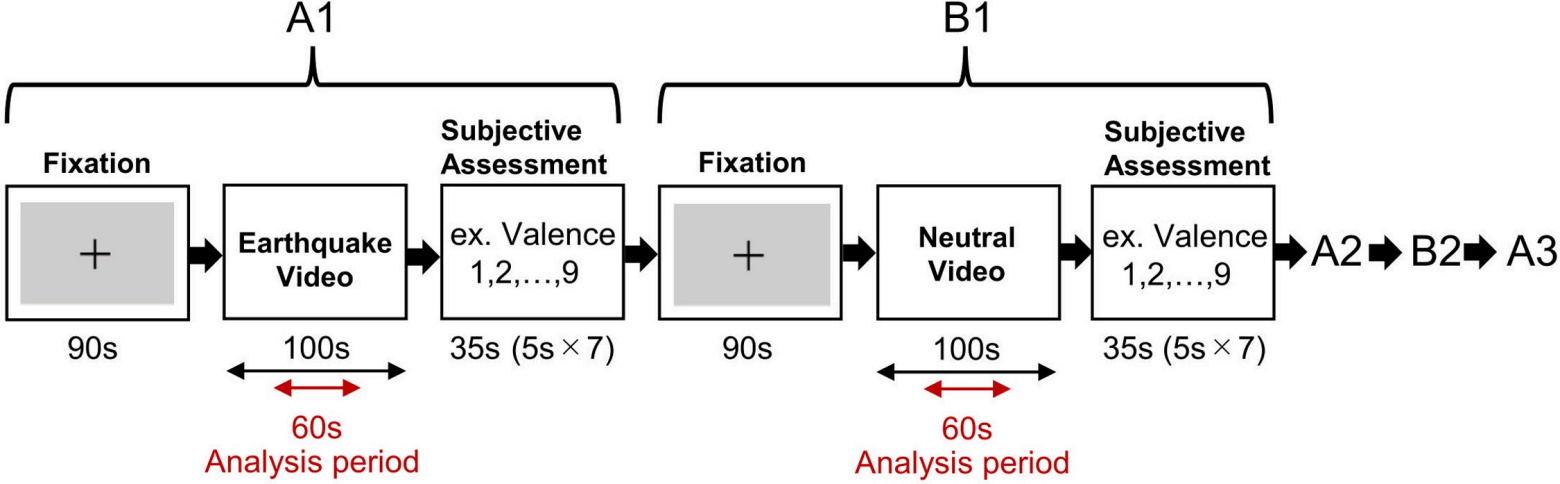
Experimental procedure of watching VR videos. Three earthquake videos and two neutral videos were shown alternately. Subjective assessments were conducted after each video presentation. The subjective assessment included seven items (valence, arousal, dominance, fear, astonishment, anxiety, and panic).

First, the participants provided their personal information and completed anxiety and personality trait rating scales. Next, the participants watched videos wearing NIRS and HMD and responded to a subjective evaluation of each video. As shown in Fig 2, a block design comprising earthquake and neutral video viewing conditions for each 100 s duration was used. The Self Assessment Manikin (SAM), a non-verbal picture-based questionnaire, was used to evaluate the participant’s feelings and emotions [11]. The subjective evaluation was conducted on a 9-point Likert scale ranging from 1 to 9 for valence, arousal, and dominance of the SAM and 7-point Likert scale ranging from 1 to 7 for fear, astonishment, anxiety, and panic.

Finally, the participants answered a questionnaire on their previous earthquake experiences that asked about the maximum earthquake intensity according to the definition of the Japan Meteorological Agency (JMA) seismic scale [12], location, emotion, and behavior. Personal information included age, sex, educational and occupational history, dominant hand, intelligence quotient (IQ) assessed using the Japanese Adult Reading Test (JART), visual acuity, and sleeping time at night. The Japanese version of the State-Trait Anxiety Inventory (STAI-JYZ: new version STAI) was used as the anxiety scale, which is a self-report questionnaire that measures two aspects: anxiety responses to anxiety-provoking events in a person’s temporary state (state anxiety) and the tendency toward anxiety in a steady state (trait anxiety). The Maudsley Personality Inventory (MPI) was used to measure personality and two basic personality characteristics: neuroticism (N scale) and extraversion-introversion (E scale) [13]. Each experiment took approximately 90 min and included an explanation.

### NIRS data acquisition

NIRS device (portable brain activity measurement device HOT-2000-VR, NeU, Japan) with two channels was used to measure CBF. The sampling rate of this device was 100 ms, consisting of a light-emitting diode (LED) with a wavelength of 810 nm, one on each side of the headset, and two sensor units (approximately 1 cm and 3 cm from the LED). The two NIRS channels were located in the bilateral dorsolateral prefrontal cortex (DLPFC, left Fp1-F7 and right Fp2-F8 using the international 10–20 method), and the top of the face cushion of the HMD and the NIRS probe were superimposed approximately 3 cm above the nasion. This device calculates the changes in total hemoglobin (delta-totalHb) in the right and left hemispheres (cerebral cortex) using a method called real-time scalp signal separation [14]. Previous studies have shown that wavelength at 810 nm is near or just an isosbestic point of oxy-Hb and deoxy-Hb *in vivo*, and thus, delta-totalHb reflects delta-oxyHb [15, 16]. Hence, in this study, the total-Hb was used as an index to reflect CBF changes. Additionally, the pulse rate (beats/min) estimated from the sensor readings was recorded.

### Data analysis

NIRS signal processing was conducted as previously described [4]. The specific methods used are described below.

#### Autonomic nervous system indices

We first performed a derivative and normalization of the signal reflecting the scalp blood flow recorded by a sensor unit positioned 1 cm away from the LED, emitting near-infrared light in the right channel of the NIRS. Next, the pulse wave peak was estimated based on the preprocessed scalp blood flow signal, and the peak-to-peak interval time was calculated. Autonomic indices, such as cardiac sympathetic index (CSI) and cardiac vagal index (CVI), were calculated using the Lorenz plot method proposed by Toichi et al. [17].

#### Cerebral blood flow change and lateral index

First, a moving average every 3 s (30 points) was applied to the total-Hb [measured values] acquired using NIRS every 0.1 s to remove high-frequency noise. Next, the trends and periodic fluctuations were removed, and the data were processed using a moving median filter as follows: Trend variations were noise due to continuous changes, such as sweating or increased temperature at the point of measurement, and periodic variations were noise due to heartbeat or respiration variations. The residual y(n) at a given time n, obtained using Eq (1), is a pure signal of the brain activity. V_trend_ (n) is a third-order polynomial approximated by minimizing the residual y(n), that is, the trend variation. V_period_ (n) means the periodic variation, which shows a sinusoidal periodic variation of delta-Hb_total_ (n) ‐ V_trend_ (n). The period of periodic variation was 1/2 of all measurement times to minimize the standard deviation of the residual y(n), which was the residual obtained by dividing the trend variation by the periodic variation.


$$\text{y}(\text{n})={\text{delta-Hb}}_{\text{total}}(\text{n})-\left\{{\text{V}}_{\text{trend}}(\text{n})+{\text{V}}_{\text{period}}(\text{n})\right\}$$ (1)

A moving median filter of 200 points was used to reduce the noise. The moving average t(n) represents the true change in total hemoglobin, and the left and right t(n) are named delta-totalHb(L) and delta-totalHb(R). The units for delta-totalHb(L) and delta-totalHb(R) are expressed in arbitrary units (a.u.) in this study because the values are the product of the unit of total hemoglobin concentration (millimol/L: mM) and the unit of optical path length (cm) of the reflected light received by the light-receiving probe. The lateral index (LI) was determined as an index of CBF asymmetry in the left and right dorsolateral prefrontal cortices based on a previous study by Ishikawa et al. [18].

### Statistical analysis

All results are presented as the mean ± standard error. First, the mean values of CSI, CVI, CVI/CSI, pulse rate, delta-totalHb(L), delta-totalHb(R), LI, and subjective evaluations (valence, arousal, dominance, fear, astonishment, anxiety, and panic) were obtained for three earthquake videos and two neutral videos. Autonomic indices, pulse rate, and change in CBF were calculated for the data during the 60 s from 20 s to 80 s after starting the 100 s video.

After confirming the normality of data using the Shapiro-Wilk test, a Wilcoxon signed-rank test with Bonferroni correction was performed to examine the differences between the seismic and neutral videos for each index. Furthermore, among the subjective ratings, emotional valence, arousal, and dominance of the SAM were tested using the Mann-Whitney U-test with Bonferroni correction with independent samples between the VR video in this study and screen images from our previous study [4]. The IBM SPSS Statistics Version 27 and College Analysis Ver. 8.4 [19] was used for analysis, and the significance level of 5 percent was Bonferroni-corrected for each subjective evaluation, physiological index, and comparison of the subjective evaluation of VR video and screen images. We used the effect size (r) of the Wilcoxon signed-rank test (paired) and the Mann-Whitney U-test (unpaired), r = 0.1 (small effect size), r = 0.3 (medium effect size), and r = 0.5 (large effect size) [20].

## Results

The following are the results of the participants’ character, a comparison of subjective evaluations, and physiological indicators between the two conditions.

### Participant characteristics

In this study, data from 21 participants (10 males and 11 females, aged 21.4 ± 0.2 years) were analyzed, with 3 of the 24 participants excluded because they had temporarily interrupted the measurement owing to physical conditions or were unable to view the video owing to strong drowsiness. Heart rate data were used for 19 participants, excluding 2 more participants owing to measurement errors. The 21 participants had an educational history of 14.8 ± 0.2 years, slept 6.5 ± 0.2 h the previous day, were right-handed, had an IQ of 108.7 ± 1.1 on the JART, and had visual acuity >0.7 in both eyes. The IES-R was 1.4 ± 0.8 points, and none of the participants were excluded owing to earthquake trauma. For the STAI scores, the scores for state anxiety and trait anxiety were 33.2 ± 1.5 and 39.0 ± 1.8, respectively; for the MPI scores, the N scale was 17.1 ± 2.5, and the E scale was 31.8 ± 2.2. When asked about the earthquakes they had actually experienced, three (14.3%) had experienced six earthquakes referring to the JMA scale, five (23.8%) had experienced a maximum intensity of 5+, and all had experienced a maximum intensity of 3 or higher, except for one respondent who was unsure. The locations where they had experienced earthquakes included bedrooms (n = 7), living rooms (n = 4), and outdoors (n = 2). In terms of feelings during the earthquake, some were astonished (n = 15), upset and confused (n = 15), fearful (n = 11), fearful (n = 10), worried (n = 6), and impatient (n = 6). In terms of behavior, some participants said that they moved away from where objects might fall (n = 6), cowered under a sturdy desk or table (n = 6), or were too frustrated or afraid to move (n = 4).

### Subjective evaluation

**Fig 3:**
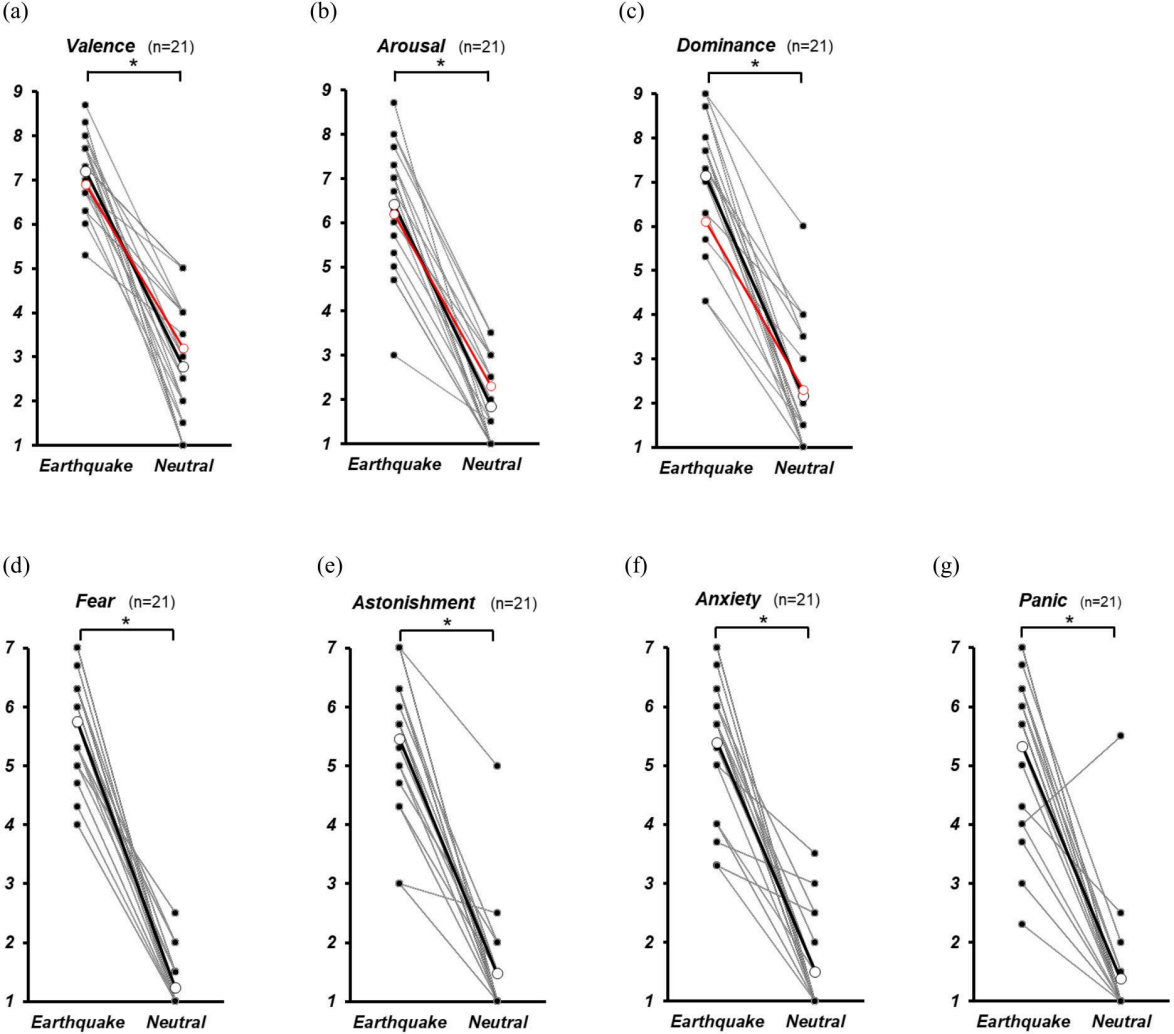
Comparison of subjective evaluation between earthquake and neutral conditions. The mean rate values of the three earthquakes or two neutral images for individual participants (filled black circles) and all participants (unfilled black circles) in the current VR condition are shown. The averages of all the participants (unfilled red circles) in the previous study [4] are also shown for (a) valence, (b) arousal, and (c) dominance. Statistical analyses were performed using Wilcoxon signed-rank tests with Bonferroni correction. *: p < 0.0071.

Individual changes in the subjective ratings (filled black circles) and means (unfilled black circles) are shown in Fig 3, and the raw scores are shown in S1 Table (columns B to AJ). Comparisons between the conditions showed that valence, arousal, dominance, fear, astonishment, anxiety, and panic were significantly higher in the earthquake condition than in the neutral condition (p < 0.001). For valence, arousal, and dominance, we compared these results with those of our previous study (unfilled red circles in Fig 3), which used a 2D video. Although no significant differences were observed, a moderate effect was observed for the dominance of earthquakes, which was higher in the VR condition than in the 2D video condition. (7.1 ± 0.3 (VR) vs. 6.1 ± 0.4 (2D video), p = 0.03, r = -0.37) (see S2 Table).

### Autonomic indices, pulse rate and cerebral blood flow changes

**Fig 4:**
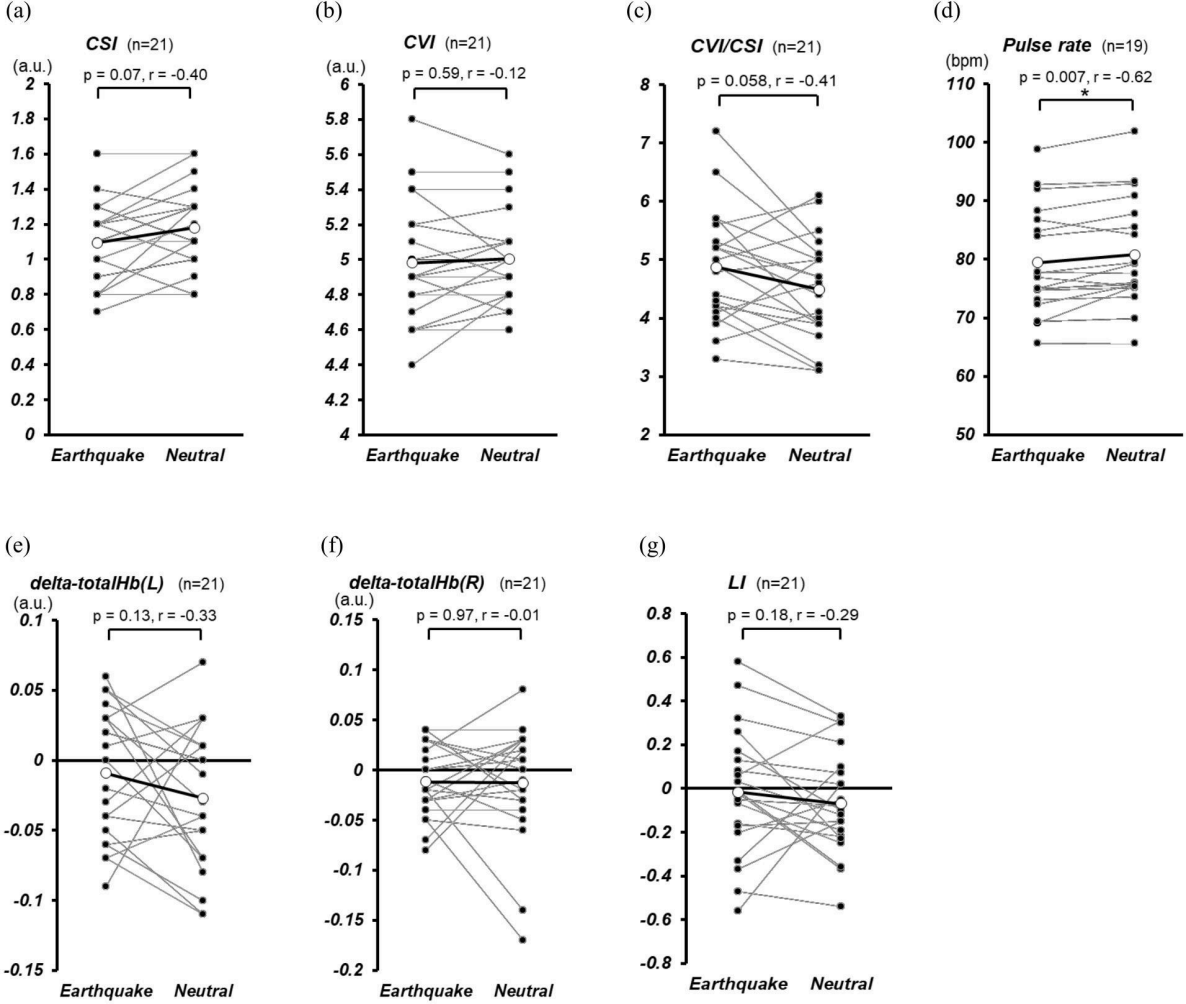
Comparison of physiological indices between earthquake and neutral conditions. The mean rate values of the three earthquakes or two neutral images for individual participants (filled circles) and all participants (unfilled circles) are shown. Statistical analyses were performed using Wilcoxon signed-rank tests with Bonferroni correction. *: p < 0.0071. CSI: cardiac sympathetic index, CVI: cardiac vagal index, delta-totalHb: the change in total hemoglobin, LI: lateral index.

The results for each physiological index are shown in Fig 4. In each figure, the means of viewing the three seismic and two neutral videos for each participant are plotted in black, with the open circles indicating the means for all participants. The values of each physiological indicator are shown in S1 Table (columns AK to BS).

Comparison by video condition showed that the pulse rate was significantly lower in the seismic condition than in the neutral condition (p = 0.007, r = -0.62, Fig 4(D)); the other indices had no significant differences. However, there was a tendency for CSI to decrease, CVI/CSI to increase, and delta-totalHb(L) to increase slightly under seismic conditions, and the effect sizes were moderate for CSI (p = 0.07, r = -0.40, Fig 4(A)) and CVI/CSI (p = 0.058, r = -0.41, Fig 4(C)) and delta-totalHb(L) (p = 0.13, r = -0.33, Fig 4(E)).

## Discussion

This study aims to investigate the capture of emotional responses during disaster experiences in an immersive and realistic VR environment. We compared physiological indices such as CBF, pulse rate, and autonomic nervous system activity while viewing earthquake and neutral videos using a NIRS system that can be worn simultaneously with an HMD.

### Subjective evaluation of earthquake vs. neutral

First, in the subjective evaluation of the earthquake and neutral videos, the earthquake videos scored significantly higher than the neutral videos in terms of emotional valence, arousal, dominance, fear, astonishment, anxiety, and panic (Fig 3). In other words, it was confirmed that the earthquake video induced more negative emotions than the neutral one.

### Physiological indices of earthquake vs. neutral

Second, in the physiological indices, the pulse rate was significantly lower when the earthquake video was viewed compared to the neutral video (Fig 4(D)). In addition, although there was no significant difference in the autonomic index, there was a trend toward a decrease in the CSI, which indicates sympathetic activity, and an increase in the CVI/CSI, which indicates parasympathetic dominance, under earthquake conditions (Fig 4(A) and 4(C)). These results support our hypothesis that pulse rate and sympathetic activity are decreased by the VR earthquake experience.

Previous studies have reported that heart rate decreases more for unpleasant auditory and visual stimuli than for pleasant stimuli [21, 22], as well as for fear of imminent threats, disgust associated with physical mutilation, and sudden sadness [23]. The finding that the VR earthquake videos evoked significantly more negative emotions than the neutral film in the present subjective rating suggests that the negative emotions evoked in the present study reduced sympathetic nerve activity and pulse rate.

Conversely, we discuss the fact that the parasympathetic index (CVI) showed little difference between the seismic and neutral conditions (Fig 4(B)). Berntson et al. reported that sympathetic and parasympathetic activities may be non-interactive and independent [24], and Kreibig reported that disgust associated with body mutilation stated that the associated decrease in pulse rate may be caused by sympathetic withdrawal rather than parasympathetic influence [23]. Therefore, we assume that the results of this study were caused by an independent decrease in the sympathetic nervous system activity due to negative emotional arousal.

In addition, the comparison between the earthquake and neutral conditions in the indices of CBF change (delta-totalHb(L), delta-totalHb(R), and LI) showed no significant differences (Fig 4(E)–4(G)), which did not support our hypothesis that CBF changes in the prefrontal cortex increase during the viewing of earthquake images. The results also did not support Marumo et al.’s report [25] that oxy-Hb in the right lateral ventral prefrontal cortex and supplementary motor cortex increased when fearful facial expressions were presented or Hoshi et al.’s report [26] that oxy-Hb in the bilateral lateral ventral prefrontal cortex increased when unpleasant pictures were presented. However, there was a slight tendency for delta-totalHb(L) to increase more in the earthquake condition than in the neutral condition (Fig 4(E)) since the right amygdala is associated with the rapid processing of emotional stimuli, while the left is associated with maintaining impulsive stimuli [27]; the present results may reflect emotional responses to sustained stimuli.

### Impact of VR videos on emotional change

Third, we compared the results of our previous report [4], in which 2D video clips were projected onto a common projection screen.

As shown in Fig 3 and S2 Table, regarding the subjective evaluation of the earthquake video, the dominance of SAM showed higher trends in the VR video than in the 2D video presentation in the previous report [4], and the mean values of emotional valence and arousal were higher than the previous time, although not significant. This suggests that the VR images in the present study were more realistic earthquake experiences than the screen images and nearly supports the hypothesis of the present study that earthquake experiences in VR space increased negative emotions more than 2D images.

We observed the same trend as in our previous study [4] for pulse rate and autonomic activity. Referring to the p-values and effect sizes, sympathetic activity and pulse rate tended to decrease more in the earthquake condition than in the neutral condition, and there was little difference in the parasympathetic response between the earthquake and neutral conditions, similar to the findings of a previous study.

Although there was no significant difference in CBF change between the seismic and neutral conditions in both the present and the previous study, the mean value of delta-totalHb(L) was higher in the seismic condition than in the neutral condition. There was almost no difference in delta-totalHb(R) between the seismic and neutral conditions, which was a common point. For delta-totalHb(L), the effect size was larger this time than in the previous study, with p = 0.13 and r = -0.33.

These results suggest that viewing earthquake videos decreases sympathetic activity and pulse rate and that immersive VR video viewing may induce greater emotional changes and increase brain activity in the left DLPFC than 2D video on screen display.

### Novelty of simultaneous measurement using NIRS and VR

Finally, we discuss the novelty of our experimental technique for NIRS measurement and immersive VR experience. There have already been several reports of NIRS measurements during VR experiences. A previous study measured brain activity using NIRS while firefighters performed a fire task in a VR environment [6], and another report measured brain activity while firefighters performed a line division bisection task using a VR helmet modified with multichannel NIRS and an HMD [7]. The present study is novel because it used NIRS to capture emotional responses during a VR disaster experience where no cognitive task was imposed. This is the first research finding obtained using NIRS and VR.

Moreover, in the questionnaire regarding individual experiences of earthquakes, some were able to take protective actions after an earthquake of intensity JMA scale 3 or higher, while others were unable to act because of surprise or fear, suggesting that there were individual differences between those who were able to take appropriate actions and those who were not. It has been reported that heart rate variability during arousal and changes in CBF in the left and right DLPFC differ depending on the degree of emotional trauma, even in disaster victims [28].

As a limitation, since this study was an exploratory investigation with a small number of participants in a specific group (healthy young adults), further validation with a larger variety of participants is needed to consider individual differences in disaster experiences and psychological trauma caused by disasters. Furthermore, the number of CBF measurements in this study was limited to two areas, at the left- and right-DLPFC, because the NIRS model that allows simultaneous attachment with a head-mounted VR system was used. The DLPFC, as well as the overall PFC area, reflects the emotional change as described by Bendall et al. [29]; however, the responses are very complex. Therefore, more studies targeting the entire brain using a multichannel-NIRS will be required to fully understand the VR-induced emotional change in humans.

## Conclusions

In this study, we investigated the capture of emotional responses in an immersive VR environment using NIRS that can be simultaneously worn with an HMD. The presentation of earthquake videos using VR evoked negative emotions subjectively and possibly enabled a more realistic earthquake experience than a 2D video using a screen. Under this VR earthquake experience, the pulse rate significantly decreased, and sympathetic nerve indices showed a decreasing trend. Furthermore, while CBF in the DLPFC did not significantly differ between the seismic and neutral conditions, there was a trend toward increased activity in the left frontal lobe in the VR environment compared to the screen-based 2D video presentation. While further investigations with a larger variety of participants are required, and modalities utilizing VR and NIRS measurements would be useful in capturing emotional responses to emotionally arousing experiences such as fear and anxiety.

## Supporting information

S1 Table Individual subjective evaluation scores and physiological index values. (XLSX)

S2 Table Comparison of subjective evaluation between VR video and 2D video conditions. Values are presented as means ± standard error. Statistical analyses were performed using the Mann-Whitney U-test with Bonferroni correction (p < 0.008). n = 21 for VR videos in the present study and n = 12 for 2D videos in the previous study [4].
(PDF)

S1 Appendix English translation of the analysis method section of Ref. 4. (PDF)
