# Supplementary material for: Capture of emotional responses under a simulated earthquake experience using near-infrared spectroscopy and virtual reality
Source: PLoS One. 2024 May 23;19(5):e0304107. doi: 10.1371/journal.pone.0304107 (PMC11115202; doi:10.1371/journal.pone.0304107)
Supplement: S2 Table — Values are presented as means ± standard error. Statistical analyses were performed using the Mann-Whitney U-test with Bonferroni correction (p < 0.008). n = 21 for VR videos in the present study and n = 12 for 2D videos in the previous study [4]. (PDF) [file pone.0304107.s002.pdf]

S2 Table. Comparison of subjective evaluation between VR video and 2D video conditions.

		**VR videos**	**2D videos**	***p*-value**	**effect size (r)**
**Earthquake**	Valence	7.2±0.2	6.9±0.2	0.37	-0.17
	Arousal	6.4±0.3	6.2±0.3	0.33	-0.17
	Dominance	7.1±0.3	6.1±0.4	0.03	-0.37
**Neutral**	Valence	2.8±0.3	3.2±0.4	0.43	0.14
	Arousal	1.8±0.2	2.3±0.2	0.15	0.26
	Dominance	2.2±0.3	2.3±0.3	0.43	0.15
