# Supplementary material for: Capture of emotional responses under a simulated earthquake experience using near-infrared spectroscopy and virtual reality
Source: PLoS One. 2024 May 23;19(5):e0304107. doi: 10.1371/journal.pone.0304107 (PMC11115202; doi:10.1371/journal.pone.0304107)
Supplement: S1 Appendix — (PDF) [file pone.0304107.s003.pdf]

S3 Appendix. English translation of the analysis method section of Ref. 4

"Otsuka H, Okahashi S, Seiyama A. Evaluation of emotional changes during earthquake video watching: A Wearable NIRS Study, Human interface: The transaction of Human Interface Society. 2022;24: 239–248. https://doi.org/10.11184/his.24.4_239. (in Japanese)"

2.6 Analysis method

2.6.1 Autonomic nervous system indices

The RR interval was estimated from the cerebral blood flow data of the HOT-2000-VR, and the autonomic index was calculated from the pulse rate variability.

Specifically, first, the signals reflecting scalp blood flow recorded by sensor unit 1, which is located 1 cm away from the LED emitting near-infrared light in the right channel of the HOT-2000-VR, were pre-processed by differentiation and standardization. Differentiation was performed by subtracting the current signal A(n) from the next signal A(n+1) in order to reduce noise due to relatively large wave trends and large movements in the signal. Standardization was performed using equation (1) in order to align the signal magnitude differences among measurement devices and individuals.

$$Standardization = \frac{signal\ (time\ n) - signal\ (the\ average\ of\ all\ signal)}{SD} \quad (1)$$

Next, the pulse wave peaks were estimated based on the preprocessed scalp blood flow signal, and the peak interval time (RR interval) was calculated. In this process, a threshold was set for the height of the standardized signal to avoid mistakenly extracting pulse wave truncations (peaks caused by the opening and closing of cardiac valves) as peaks.

Autonomic indices were calculated using the Lorenz plot method proposed by Toichi et al. A Lorenz plot is a plot of the nth RR interval in x-coordinate and the n+1th RR interval in y-coordinate on the x-y plane, and the sympathetic function index CSI (L/T) and the parasympathetic function index CVI (log10(L×T)) are calculated[19].

2.6.2 Cerebral blood flow and Lateral Index

The trend and periodic variations were removed from the left and right Δtotal-Hb [measured values] acquired by HOT-2000-VR, and the moving median filtered left and right values were obtained as Δtotal-Hb(L) and Δtotal-Hb(R) that reflect true brain activity, respectively. The details are described below.

Trend fluctuations are noise associated with persistent changes such as sweating or temperature increase at the measurement site, while periodic fluctuations are noise due to heartbeat or respiration fluctuations. To eliminate these noises, the residual y(n) at time n was obtained as the true brain activity signal using equation (2). Note that Vtrend (n) is a third-order polynomial approximated so that the residual y(n) is minimized, meaning the trend variation. Vperiod (n) is a sinusoidal waveform that extracts the periodic variation of ΔHbtotal (n) - Vtrend (n), meaning periodic variation. To minimize the standard deviation of the residual y(n) obtained by dividing the periodic variation by the trend variation, the periodic variation was defined as 1/2 of all measurement times.

$$y(n) = \Delta Hb_{total}(n) - \{ V_{trend}(n) + V_{period}(n) \} \quad (2)$$

In addition, a moving median filter was applied to reduce aperiodic noise caused by the subject's body movements and measurement errors, and the moving average t(n) was obtained using equation (3).

$$t(n) = \text{median} \{ y(n-k), \ldots, y(n), \ldots, y(n+k) \} \quad (3)$$

k is the number of terms in the moving median filter, and is set to 200, where the mean square of the difference between y(n) and t(n) satisfies the conditions of reducing noise and minimizing distortion of the original signal y(n). y(n) is the residual of each side every 0.1 second, calculated using equation (2).

The t(n) obtained by the above noise processing was again set as the true change in total hemoglobin, and was designated as Δtotal-Hb(L) and Δtotal-Hb(R) for the left and right hemoglobin, respectively. The units of Δtotal-Hb(L) and Δtotal-Hb(R) are the product of the unit of total hemoglobin concentration (millimol/L: mM) and the unit of optical path length (cm) of the reflected light received by the light receiving probe.

Next, the Lateral Index (LI) was calculated as an index of cerebral blood flow asymmetry in the left and right dorsolateral prefrontal cortices using equation (4) from the Δtotal-Hb(L) and Δtotal-Hb(R) values, referring to a previous study by Ishikawa et al.

$$LI = \frac{\{\Delta total\ L(n) - \Delta total\ L(min)\} - \{\Delta total\ R(n) - \Delta total\ R(min)\}}{\{\Delta total\ L(n) - \Delta total\ L(min)\} + \{\Delta total\ R(n) - \Delta total\ R(min)\}} \quad (4)$$

Δtotal L(n) and Δtotal R(n) are the Δtotal-Hb(L) and Δtotal-Hb(R) values every 0.1 s, respectively. When LI is positive, the left dorsolateral prefrontal cortex is more active than the right, and when LI is negative, the right dorsolateral prefrontal cortex is more active than the left.
